# Impact of multi-drug resistance on clinical outcomes of dogs with corneal ulcers infected with *Staphylococcus pseudintermedius*

**DOI:** 10.3389/fvets.2022.1083294

**Published:** 2022-11-24

**Authors:** Ashley N. Mauer, Rachel A. Allbaugh, Amanda J. Kreuder, Lionel Sebbag

**Affiliations:** ^1^Department of Veterinary Clinical Sciences, College of Veterinary Medicine, Iowa State University, Ames, IA, United States; ^2^Department of Veterinary Microbiology and Preventive Medicine, College of Veterinary Medicine, Iowa State University, Ames, IA, United States; ^3^Koret School of Veterinary Medicine, The Hebrew University of Jerusalem, Rehovot, Israel

**Keywords:** bacterial keratitis, canine, corneal ulceration, clinical outcome, multi-drug resistance, risk factors, *Staphylococcus* spp.

## Abstract

**Objective:**

Compare characteristics and clinical outcomes of dogs with infectious keratitis from *Staphylococcus pseudintermedius* considered to be multidrug-resistant (MDR) or not.

**Procedures:**

*Staphylococcus pseudintermedius* isolated as the primary pathogen from canine patients with ulcerative keratitis were considered MDR if resistant to at least one agent in three or more classes of antibiotics. Medical records were reviewed for history, patients' characteristics, clinical appearance, therapeutic interventions, and clinical outcomes.

**Results:**

Twenty-eight dogs (28 eyes) were included. Compared to non-MDR cases, MDR diagnosis was significantly more common in dogs with recent (≤30 days) anesthesia (7/15 vs. 1/13, *P* = 0.038) and more common in non-brachycephalic dogs (8/15 vs. 2/13, *P* = 0.055). Clinical appearance (ulcer size/depth, anterior chamber reaction, etc.) did not differ significantly between groups (*P* ≥ 0.055). Median (range) time to re-epithelialization was longer in MDR vs. non-MDR eyes [29 (10–47) vs. 22 (7–42) days] but the difference was not significant (*P* = 0.301). Follow-up time was significantly longer in dogs with MDR keratitis [47 (29–590) vs. 29 (13–148) days, *P* = 0.009]. No other significant differences were noted between MDR and non-MDR eyes in regard to time for ulcer stabilization [4 (1–17) days vs. 4 (1–12), *P* = 0.699], number of eyes requiring surgical stabilization (7/15 vs. 7/13, *P* = 0.246) or enucleation (1/15 vs. 2/13, *P* = 1.000), success in maintaining globe (14/15 vs. 11/13, *P* = 0.583) or success in maintaining vision (12/15 vs. 10/13, *P* = 1.000).

**Conclusions:**

MDR infections may prolong corneal healing time but did not appear to affect overall clinical outcomes in dogs with bacterial keratitis. Further research is warranted in a larger canine population and other bacterial species.

## Introduction

Bacterial keratitis is a major global cause of ocular discomfort and visual impairment in dogs and other species (1, 2). Following an injury to the eye from trauma or other causes, corneal wounds have a high tendency for infection given the presence of indigenous microflora on the corneal and conjunctival surfaces, including *Staphylococcus* species, *Streptococcus* species, *Pseudomonas* species, and gram-positive bacilli. More specifically, *Staphylococcus pseudintermedius* represents the most common bacteria causing infectious keratitis in many geographical locations around the world including North America (3–5), South America (6), Europe (7, 8), and Asia (9, 10). A component of normal flora in canines, *Staphylococcus pseudintermedius* is increasingly recognized as a major pathogen in various body systems of dogs including eyes, skin, ears, respiratory tract, reproductive tract, and urinary tract (11, 12). *Staphylococcus pseudintermedius* is also an emerging pathogen in humans (13) with reported transmission from infected dogs to the home environment and owners (14), as well as cross-contaminations to other veterinary patients in clinical settings (15).

An alarming, escalating trend of resistant *Staphylococcus pseudintermedius* strains has been described in the human and veterinary literature, including methicillin-resistant strains (MRSP) (10, 16, 17) and multidrug-resistant strains (MDR) (5, 18), that is, bacteria with “acquired resistance to at least one agent in three or more antimicrobial categories” (19). In a recent canine study, MDR represented 20% of all corneal isolates (including 33% of all *Staphylococcus* spp.) with an incidence that increased from 5% (2016) to 34% (2020) during the period examined (5). The presence of resistant bacterial strains is known to affect clinical outcomes in human patients with infectious keratitis, but to the authors' knowledge, the same has not been evaluated in dogs. In humans, corneal infections from relatively ciprofloxacin-resistant bacteria (i.e., higher minimal inhibitory concentrations) were shown to respond more slowly to therapy (20), while corneal infections caused by relatively moxifloxacin-resistant bacteria resulted in larger corneal scars (21).

The main purpose of this study was to compare the clinical outcomes of canine eyes infected by *Staphylococcus pseudintermedius* isolates classified as MDR or non-MDR. A secondary objective was to identify risk factors for dogs to develop infectious keratitis involving resistant *Staphylococcus pseudintermedius* strains.

## Materials and methods

### Inclusion and exclusion criteria

A retrospective review of electronic medical records (March 2014 to May 2020) was performed searching for dogs diagnosed with culture-confirmed bacterial keratitis (*Staphylococcus pseudintermedius*) at Iowa State University's Lloyd Veterinary Medical Center (ISU-LVMC). Cases were excluded if they involved the identification of multiple potential pathogens on culture, or if there was incomplete clinical data in the patient's medical records. Cases that presented prior to March 2014 were not included in our study as an ophthalmic-specific susceptibility profile was not available at that time.

### Ophthalmic examination

At the initial visit and subsequent rechecks, each canine patient underwent a complete ophthalmic examination by a board-certified veterinary ophthalmologist or an ophthalmology resident, including: neuro-ophthalmic assessment (menace response, dazzle reflex, pupillary light reflexes, palpebral reflex), slit lamp biomicroscopy (SL-17; Kowa Company, Ltd., Tokyo, Japan), indirect ophthalmoscopy (Keeler Vantage; Keeler Instruments, Inc., Broomall, PA, USA), rebound tonometry (TonoVet; Icare Finland Oy, Espoo, Finland), Schirmer tear test-1 (STT-1; Eye Care Product Manufacturing LLC, Tucson, AZ, USA), and fluorescein staining (Flu-Glo, Akorn, Inc., Buffalo Grove, IL, USA).

### Sample collection, bacterial isolation, and identification

At the ISU-LVMC, samples were collected with sterile culturette swabs (BBL™ CultureSwab™, Becton Dickinson and Company, Sparks, MD) that were pre-moistened with one drop of sterile saline prior to contact with the corneal wound. Samples were submitted to the Iowa State University Veterinary Diagnostic Laboratory (ISU-VDL) and processed for standard aerobic microbiologic assessment using tryptic soy agar with 5% sheep blood (blood agar) and MacConkey medium. The blood agar was incubated at 35 ± 2°C with 5–10% CO_2_ for a total length of 4 days while the MacConkey agar was incubated 35 ± 2°C without CO_2_ for a total length of 2 days. Both agar plates were observed for growth every 24 h. Organisms were then identified using Matrix-Assisted Laser Desorption Ionization Time-of-Flight mass-spectrometry (MALDI-TOF MS, Bruker).

### Antimicrobial susceptibility testing

Minimum inhibitory concentration (MIC) susceptibility testing was performed using an automated broth microdilution system (Sensititre AIM, Trek Diagnostic System Inc.) and the following susceptibility panels from Thermo Fischer Scientific: JOEYE2 (ophthalmic panel, used 2015–2020), COMPAN1F (systemic panel, used 2015–2016) and COMPGP1F (systemic panel, used 2017–2020).

The MIC data was then used to establish clinical interpretations for each isolate/antibiotic (susceptible, intermediate, and resistant) based on canine breakpoints for systemic antimicrobials (22) or, when not available, extrapolated from human clinical breakpoints for systemic antimicrobials (23, 24) following veterinary guidelines for appropriate data extrapolation (Supplementary Table 1) (25). While recommendations from Sweeney et al. (26) were considered for veterinary MDR definition based on available canine-specific breakpoints, MDR recommendations for *Staphylococcus* spp. from Magiorakos et al. (19) were utilized due to the drugs tested on the panels used and the more recent CLSI VET09 guidance, which allows for use of extrapolated human breakpoints in dogs for multiple additional antibiotics. Then, each *Staphylococcus pseudintermedius* isolate was classified as MDR (or not) if checking one or both criteria as follows:

(i) Oxacillin resistance (23): Oxacillin resistance is used to test for resistance to methicillin, which is typically conferred through acquisition of the *mecA* gene; methicillin resistance is considered to also predict resistance to all subclasses of beta-lactam antibiotics including penicillinase labile and stabile penicillins, first and third generation cephalosporins, and carbapenems.
(ii) Acquired resistance to at least one agent in three or more antimicrobial categories (19). Of note, although MICs for two polypeptides (bacitracin and polymyxin) were also described in the susceptibility profiles, these antibiotics were not included in the MDR classification given the lack of clinical breakpoints reported in human or veterinary guidelines. Therefore, MDR status was determined based on demonstrated resistance to the following drug classes in addition to beta-lactams: aminoglycosides, phenicols, fluoroquinolones, lincosamides, tetracyclines, macrolides, ansamycins, folate pathway antagonists, and glycopeptides.

### Medical records review

Using ISU-LVMC's electronic medical records system, multiple parameters were recorded for each case. Patient characteristics included: breed, age, sex, skull conformation, concurrent ocular disease, systemic disease, history of ocular trauma, recent anesthesia (within 30 days), number of medications prior to referral, number of eyedrops per day prior to referral, and duration of clinical signs prior to diagnosis. Clinical findings included: affected eye(s), Schirmer tear test-1 results, corneal ulcer location/size/depth, presence/absence of corneal cellular infiltrates or hypopyon, and aqueous flare grade. Clinical outcomes included: number of medications prescribed, number of eyedrops administered per day, number of susceptible infections to empirical antibiotic(s) prescribed at initial visit, systemic antibiotic use, time to corneal ulcer stabilization, time to ulcer re-epithelialization, time to discontinuing topical medications, whether hospitalization for intensive medical management was required, whether surgical stabilization was required (and if so, time from presentation to surgical stabilization), whether an end-stage procedure was required, number of follow-up days, corneal stromal thinning at last visit, success in maintaining globe, and success in maintaining vision (intact menace response at last follow up visit).

### Data analysis

Normality of the data was assessed with the Shapiro–Wilk test. Statistical comparison of canine eyes affected by *Staphylococcus pseudintermedius* considered MDR vs. non-MDR was performed with Mann-Whitney tests for continuous data (e.g., Schirmer tear test, number of medications or eyedrops per day, etc.) and Fisher's exact tests for categorical data (e.g., sex, skull conformation, affected eye, etc.). Statistical analysis was performed with SigmaPlot 14.5 (Systat software, Point Richmond, CA), and *P*-values < 0.05 were considered statistically significant.

## Results

Data was not normally distributed (*P* ≤ 0.047) therefore results are presented as median and range (minimum-maximum). Overall *n* = 124 isolates of *Staphylococcus pseudintermedius* were identified, but 96 isolates were excluded from further analysis owing to the presence of other potential pathogens or incomplete medical records. As such, a total of 28 eyes (16 left eyes, 12 right eyes) involving 28 canine patients were included, with a median (range) age of 8 years (1–15 years), 11 spayed female and 17 neutered male dogs. Of the 28 eyes, 8 cases were determined to be oxacillin-resistant, 7 were resistant to three or more classes of antibiotics, and 13 cases were either susceptible to all antibiotics tested or resistant to one class of antibiotics. Breeds represented in this study included Shih Tzu (*n* = 7), Boston Terrier (*n* = 4), Chihuahua (*n* = 2), French Bulldog (*n* = 2), Maltese (*n* = 2), Pomeranian (*n* = 2), Pug (*n* = 2), mixed breed (*n* = 2), and one dog of each: Cavalier King Charles Spaniel, Cocker Spaniel, Dachshund, Miniature Pinscher, and Yorkshire Terrier.

### Patient characteristics

A history of recent general anesthesia (<30 days) was significantly more common in dogs diagnosed with MDR vs. non-MDR bacterial keratitis (46.7 vs. 7.7%, respectively; *P* = 0.038). Further, non-brachycephalic breeds were more commonly affected by MDR vs. non-MDR bacterial keratitis (53.3 vs. 15.4%), although this difference was not statistically significant (*P* = 0.055). Before referral, dogs diagnosed with MDR bacterial keratitis received a greater number of medications and daily eye drops when compared to non-MDR cases [4 (1–6) vs. 2 (0–10) and 14 (2–16) vs. 6 (0–53), respectively], although these differences were not statistically significant (*P* ≥ 0.095). Other parameters pertinent to “patient characteristics” are summarized in Table 1.

**Table 1:** Summary of selected characteristics from 28 canine patients and associated corneal ulcers infected with *Staphylococcus pseudintermedius*.

	**MDR**	**Non-MDR**	***P-*value**
Age (years)	8 (4–13)	9 (4–15)	0.561
Sex (number of females)	8/15	3/13	0.137
Sex (number of males)	7/15	10/13	0.137
Cephalic conformation (number of brachycephalic dogs)	7/15	11/13	0.055
Concurrent systemic disease (number of eyes)	7/15	5/13	0.718
Concurrent ocular disease (number of eyes)	11/15	7/13	0.433
History of ocular trauma (number of eyes)	5/15	3/13	0.686
History of anesthesia (number of eyes)	7/15	1/13	0.038
Number of medications prior to referral	4 (1–6)	2 (0–10)	0.095
Number of eye drops prior to referral	14 (2–16)	6 (0–53)	0.165
Duration of signs (days)	5 (1–34)	7 (1–37)	0.938

Results are presented as median (range) for numerical data and proportion of dogs for categorical data, comparing dogs with MDR vs. non-MDR bacterial keratitis with Mann-Whitney and Fisher's exact tests (P-values < 0.05 considered significant).

MDR, Multidrug resistant.

### Clinical findings

Median depth of corneal ulcers was smaller in MDR vs. non-MDR bacterial keratitis (50 vs. 78%) but this difference was not statistically significant (*P* = 0.065). Other parameters pertinent to “clinical findings” are summarized in Table 2.

**Table 2:** Summary of clinical findings from 28 canine patients and associated corneal ulcers infected with *Staphylococcus pseudintermedius*.

	**MDR**	**Non-MDR**	***P-*value**
Affected eye (number of right eyes)	8/15	4/13	0.276
STT-1 (mm/min)	13 (2–24)	17 (0–33)	0.150
Location of ulcer (number of central ulcers)	9/11	8/11	1.000
Ulcer size (mm^2^)	18 (5–100)	16 (1–35)	0.470
Ulcer depth (%)	50 (10–100)	78 (20–100)	0.065
Corneal WBC infiltrates (number of eyes)	3/4	7/10	1.000
Hypopyon (number of eyes)	2/4	6/11	1.000
Aqueous flare	2 (0–3)	2 (0–4)	0.212

Results are presented as median (range) for numerical data and proportion of dogs for categorical data, comparing dogs with MDR vs. non-MDR bacterial keratitis with Mann-Whitney and Fisher's exact tests (P-values < 0.05 considered significant).

STT-1, Schirmer tear test-1; MDR, Multidrug resistant; WBC, white blood cells.

### Clinical outcomes

Groups did not differ in the number of eyes for which the corneal infection was considered susceptible to the empirical antibiotic(s) prescribed at the initial visit (*P* = 0.102). No significant differences were noted between both groups (MDR vs. non-MDR) in the need for end-stage procedure such as enucleation (6.7 vs. 15.4%, respectively; *P* = 1.000), nor in the likelihood of success in maintaining globe (93.3 vs. 84.6%, respectively; *P* = 0.583) or maintaining vision (80 vs. 76.9%, respectively; *P* = 1.000). Further, median follow-up time was significantly longer in dogs with MDR vs. non-MDR bacterial keratitis (47 vs. 29 days, *P* = 0.009). Other parameters pertinent to “clinical outcomes” are summarized in Table 3.

**Table 3:** Summary of therapeutics and clinical outcomes from 28 canine patients and associated corneal ulcers infected with *Staphylococcus pseudintermedius*.

	**MDR**	**Non-MDR**	***P-*value**
Number of topical and systemic medications prescribed	4 (3–5)	4 (2–7)	0.351
Number of eye drops per day	22 (12–61)	20 (11–55)	0.800
Number of susceptible infections to empirical antibiotic(s) prescribed at initial visit	11/15	13/13	0.102
Systemic antibiotics used (number of eyes)	7/15	7/13	1.000
Time to stabilization (days)	4 (1–17)	4 (1–12)	0.699
Time to re-epithelialization (days)	29 (10–47)	22 (7–42)	0.301
Time to discontinue topical medications (days)	29 (15–330)	29 (13–148)	0.750
Surgical stabilization (number of eyes)	7/15	7/13	0.246
Time to surgical stabilization (days)	0 (0–3)	0 (0–45)	0.927
End-stage procedure (number of eyes)	1/15	2/13	1.000
Follow-up (days)	47 (29–590)	29 (13–148)	0.009
Stromal thinning last visit (%)	20 (0–100)	50 (0–100)	1.000
Success maintaining globe (number of eyes)	14/15	11/13	0.583
Success maintaining vision (number of eyes)	12/15	10/13	1.000

Results are presented as median (range) for numerical data and proportion of dogs for categorical data, comparing dogs with MDR vs. non-MDR bacterial keratitis with Mann-Whitney and Fisher's exact tests (P-values < 0.05 considered significant).

MDR, Multidrug resistant.

## Discussion

Resistant infections from *Staphylococcus pseudintermedius* represent a serious challenge in health care for human and veterinary patients (12, 27). The virulence of *Staphylococcus pseudintermedius* can be explained by several factors such as enzyme secretion (e.g., coagulase, thermonuclease, and proteases), toxin secretion (e.g., exfoliative toxin, enterotoxin, and cytotoxins) (28), and biofilm formation at the infection site (29). Specific to dogs with bacterial keratitis, a recent study showed that the biofilm-formation ability and rates of virulence gene carriage and antibiotic resistance were higher in the isolates of *Staphylococcus pseudintermedius* from dogs with keratitis compared with those from the healthy dogs (30). In humans, the reported detrimental effects of resistant strains include higher morbidity and mortality rates (31), limited antimicrobial treatment options for physicians, prolonged hospitalization times and increased healthcare costs (32, 33). Specific to the field of ophthalmology, corneal infections from bacteria with higher MICs were shown to cause prolonged healing time and greater scar formation (20, 21). In veterinary medicine, a similar trend toward resistant bacterial isolates has been reported in the last two decades (34–36), although the actual impact on clinical outcomes of veterinary patients is poorly understood.

In the present study, no noticeable differences were noted in the clinical appearance of bacterial keratitis from MDR or non-MDR strains of *Staphylococcus pseudintermedius* in dogs. Specifically, there were no significant discrepancies in the location, size or depth of the ulcer, presence/absence of corneal infiltrates or severity of the anterior chamber reaction (aqueous flare and hypopyon). In the absence of clinical cues, MDR strains can only be recognized by collecting samples for culture and susceptibility testing, however results are often delayed by several days to weeks. Clinicians could also increase their level of suspicion for MDR strains by identifying risk factors in their canine patients. Here, a major risk factor for MDR infections was recent anesthesia prior to developing corneal ulceration, possibly related to anesthesia-associated reductions in lacrimation (37)—lowering corneal protection and levels of natural defenses in the tear film (e.g., antimicrobial peptides)—as well as the presence of environmental contaminants in the vicinity of the anesthetized dog that could result in nosocomial infection (15). Another risk factor for MDR infections is a higher number of daily ophthalmic medications/eyedrops prior to referral, likely owing to natural selection of resistant bacteria from repeated antibiotic use (38) and the influence of corticosteroid use on bacterial keratitis (39, 40). Of note, recent anesthetic event and prior topical therapy were also described as predisposing factors for infectious keratitis in previous canine studies (39, 40), but the present study further emphasizes the potential for *resistant* infections with these two risk factors. Interestingly, while brachycephalic dogs are often considered at higher risk to develop corneal ulceration when compared to non-brachycephalic dogs (39–41), the present study showed a non-significantly higher rate of MDR infections in corneas of non-brachycephalic dogs. This finding is consistent with a recent report by Hewitt et al. who showed that two non-brachycephalic breeds (Pomeranian, Saint Bernard) were significantly more likely to yield a MDR isolate when compared to mixed breed dogs (5).

Ultimately, MDR diagnosis in dogs with bacterial keratitis did not seem to influence the overall clinical outcome, with no significant differences between MDR and non-MDR eyes in terms of successful vision-preservation or successful globe-preservation. Nonetheless, time to re-epithelialization and follow-up times were longer in dogs with MDR infections, two findings that possibly translate into more labor intensive treatment and increased financial burden from the owners' perspective. On the same note but in another clinical field (dermatology), the outcome of dogs with skin infections did not differ whether the responsible pathogen (*Staphylococcus pseudintermedius*) was methicillin-resistant or methicillin-susceptible, although some cases of MRSP pyoderma took longer to treat (42). In ophthalmology and dermatology, antibiotics can be administered directly at the site of infection, providing higher concentrations than ones that which can be achieved systemically. All of the clinical breakpoint interpretations used in this study were for systemic antimicrobial use; there are no clinical breakpoints that have been established for topical antibiotic use in humans or animals. Because of this, CLSI does not recommend the routine use of AST to determine topical therapies. The higher concentrations achieved with topical therapy could be therapeutic *in vivo* despite antimicrobial resistance demonstrated *in vitro*, but further work is needed to support this hypothesis. In a recent canine study, one third of corneal ulcers infected with fluoroquinolone-resistant β*-hemolytic Streptococcus* achieved a good outcome despite initial treatment with a topical fluoroquinolone (40).

Importantly, the authors continue to strongly advise corneal culture and susceptibility testing for canine patients with suspected infectious keratitis. First, corneal cultures help identify the causative pathogen and therefore provide clinicians with valuable insight on drug(s) of choice and clinical prognosis. Second, MDR identification provides critical data for healthcare professionals to reduce the risk of nosocomial infections (e.g., disinfection and sterilization of contact areas) (15) and to ensure surveillance of any emergent antibacterial resistance. Last, owners of canine patients with MDR strains should ideally be kept informed given the zoonotic potential of *Staphylococcus pseudintermedius* (13, 14).

The present study was limited to cases where *Staphylococcus pseudintermedius* was determined to be the only primary pathogen identified to reduce variability and better focus on the variable of interest (i.e., MDR status). However, results of this work cannot be extrapolated to canine patients with polymicrobial infections (up to 21% of cases) (3, 43) or dogs with infectious keratitis from *Streptococcus* spp., *Pseudomonas* spp., or other common bacterial pathogens reported for this condition. A recent study by Tsvetanova et al. (40) showed that melting corneal ulcers associated with *Pseudomonas* spp. were significantly more likely to result in globe loss than other bacterial pathogens (40), and it would be interesting to know whether MDR further complicated clinical outcomes or not. Owing to the retrospective nature of the work, another study limitation is the lack of information on other factors that may have influenced clinical outcomes, such as owner compliance, albumin levels in tear film (44), or virulence of specific bacterial strains (45).

In conclusion, resistant strains of *Staphylococcus pseudintermedius* did not appear to negatively impact clinical outcomes in dogs with bacterial keratitis, although treatment duration and healing time may be prolonged when compared to non-resistant strains.

## Data availability statement

The raw data supporting the conclusions of this article will be made available by the authors, without undue reservation.

## Ethics statement

Ethical review and approval was not required for the animal study because the work was a retrospective analysis of bacterial cultures collected in dogs as part of the patients' standard of care. Written informed consent for participation was not obtained from the owners because the work was a retrospective analysis of bacterial cultures collected in dogs as part of the patients' standard of care.

## Author contributions

LS conceptualized and designed the study in consultation with AM, AK, and RA. AM and LS performed the experiments. LS and AK analyzed the data. All authors wrote the manuscript. All authors contributed to the article and approved the submitted version.

## Conflict of interest

The authors declare that the research was conducted in the absence of any commercial or financial relationships that could be construed as a potential conflict of interest.

## Publisher's note

All claims expressed in this article are solely those of the authors and do not necessarily represent those of their affiliated organizations, or those of the publisher, the editors and the reviewers. Any product that may be evaluated in this article, or claim that may be made by its manufacturer, is not guaranteed or endorsed by the publisher.
