# Supplementary material for: Impact of multi-drug resistance on clinical outcomes of dogs with corneal ulcers infected with Staphylococcus pseudintermedius
Source: Front Vet Sci. 2022 Nov 24;9:1083294. doi: 10.3389/fvets.2022.1083294 (PMC9729527; doi:10.3389/fvets.2022.1083294)
Supplement: Supplementary file 1 [file Table_1.pdf]

Supplementary Table 1. Interpretive categories and minimum inhibitory concentration breakpoints (in μg/mL) based on Performance Standards for Antimicrobial Disk and Dilution Susceptibility Tests for Bacteria Isolated from Animals, 5th Edition (CLSI, Vet01S).

Antimicrobial agent	Susceptible	Intermediate	Resistant	Breakpoint source
Aminoglycosides				
Amikacin	≤ 4	8	≥ 16	Canine systemic*
Gentamicin	≤ 4	8	≥ 16	Human systemic
Phenicols				
Chloramphenicol	≤ 8	16	≥ 32	Human systemic
Fluoroquinolones				
Difloxacin	≤ 0.5	1-2	≥ 4	Canine systemic
Enrofloxacin	≤ 0.5	1-2	≥ 4	Canine systemic
Marbofloxacin	≤ 1	2	≥ 4	Canine systemic
Orbifloxacin	≤ 1	2-4	≥ 8	Canine systemic
Pradofloxacin	≤ 0.25	0.5-1	≥ 2	Canine systemic
Lincosamides				
Clindamycin	≤ 0.5	1-2	≥ 4	Canine systemic
Tetracyclines				
Doxycycline	≤ 0.12	0.25	≥ 0.5	Canine systemic
Minocycline	≤ 0.5	1	≥ 2	Canine systemic
Tetracycline	≤ 0.25	0.5	≥ 1	Canine systemic
Macrolides				
Erythromycin	≤ 0.5	1-4	≥ 8	Human systemic
Ansamycins				
Rifampin	≤ 1	2	≥ 4	Human systemic
Folate pathway antagonists				
Trimethoprim sulphamethoxazole	≤ 2/38	--	≥ 4/76	Human systemic
Beta-lactams				
Oxacillin	≤ 0.25	--	≥ 0.5	Human systemic
Penicillin	≤ 0.12	--	≥ 0.25	Human systemic
Glycopeptides				
Vancomycin	≤ 4	8-16	≥ 32	Human systemic

* Not utilized due to plate configuration issues (testing not in range)
